# Damage to the right temporoparietal junction, but not lateral prefrontal or insular cortex, amplifies the role of goal-directed attention

**DOI:** 10.1038/s41598-018-36537-3

**Published:** 2019-01-22

**Authors:** Elena Pedrazzini, Radek Ptak

**Affiliations:** 1 Laboratory of Cognitive Neurorehabilitation, Faculty of Medicine, University of Geneva, Geneva, Switzerland; 2 Division of Neurorehabilitation, Geneva University Hospital, Geneva, Switzerland; 3 Faculty of Psychology and Educational Sciences, University of Geneva, Geneva, Switzerland

## Abstract

Whether an object captures attention depends on the interplay between its saliency and current behavioral predispositions of the observer. Neuroimaging work has implied a ventral attention network, comprising the temporoparietal junction (TPJ), lateral prefrontal cortex (lPFC) and the insula, in attentional orienting toward salient events. Activity of the TPJ is driven by novel and unexpected objects, while the lateral prefrontal cortex is involved in stimulus-driven as well as goal-directed processing. The insula in turn, is part of a saliency network, which has been implicated in detecting biologically salient signals. These roles predict that damage to the TPJ, lPFC, or insula should affect performance in tasks measuring the capture of attention by salient and behaviorally relevant events. Here, we show that patients with lesions to the right TPJ have a characteristic increase of attentional capture by relevant distracters. In contrast, damage to the lPFC or insular cortex only increases reaction times, irrespective of the task-relevant properties of distracters. These findings show that acquired damage to the TPJ pathologically amplifies the capture of attention by task-relevant information, and thus indicate that the TPJ has a decisive role in goal-directed orienting.

## Introduction

Objects in our environment may capture attention because of their physical characteristics, or because of our behavioral predispositions^1,2^. Priority-based theories of attention contend that subjective biases and sensory properties interactively set the criterion that will guide attentional selection^3–5^. This view entails that the subjective value of a stimulus depends on current behavioral goals and how they combine or compete with sensory signals to adjust attentional priority^6–8^.

Substantial functional imaging evidence relates the integration of behavioral goals and stimulus-driven biases to the interaction between a bilateral dorsal and a right-lateralized ventral attention network^9,10^. The dorsal network exhibits almost ubiquitous activation in many attention paradigms, whether performance is in a controlled or an automatic mode, and whether it involves covert or overt attention shifts^11–14^. Activation patterns of the ventral stream, which embodies the temporoparietal junction (TPJ), lateral prefrontal cortex (lPFC) and insular cortex, are less consistent. The right TPJ shows transient changes of activity in tasks requiring the reorienting of attention toward a target appearing at an unexpected location^15–17^. An influential proposal is therefore that the right TPJ reacts to sudden and improbable stimuli by interrupting and resetting activity of the dorsal attention network^10^. However, this hypothesis predicts a faster onset of activation of the TPJ than regions of the dorsal attention network^18^, which is not supported by neurophysiological findings. Single-unit studies show that neurons in the lateral intraparietal cortex and the frontal eye fields are activated already 45–60 ms following the sudden onset of a stimulus^19–21^, which is substantially earlier than inferior parietal cortex (~90 ms)^22^. In humans, the N2pc is an evoked potential associated with shifts of attention which appears at 180 ms over the dorsal parietal lobe^23^, and even earlier activity has been measured to sudden peripheral onsets^24^. In contrast, the activation of the TPJ is more compatible with the P3, a potential that appears at 300–400 ms and is modulated by expectations, target probability and representations about the task maintained in working memory^25^. Though its neural sources are widely distributed across frontal, temporal and parietal cortex^26^, damage to the TPJ significantly reduces the P3 potential, which is not observed after frontal or superior parietal damage^27^. In addition the TPJ is not only involved in attention, but is also activated when subjects search for information in memory or make inferences about other people^10,28,29^. Moreover, this region is also strongly involved when participants reason about other people’s mental states^30^ or when they are required to adapt their expectations to changing task contingencies^18^. These observations support a multimodal role of the TPJ in focalizing attention on and updating representations about internal or external behaviorally relevant events, be it in the attention, memory, or social domain.

The contribution of the anterior ventral attention network to the orienting of attention is less well defined. A functional connectivity study found that the right lPFC is coupled to the dorsal and ventral attention networks, suggesting a possible role as integrator of goal-directed and stimulus-driven attention^31^. This interpretation is supported by the finding that during visual search the lPFC shows activity consistent with goal-oriented processing, but also reacts upon the appearance of salient surprise stimuli^32^. Having access to stimulus-driven as well as goal-directed signals the right lPFC is thus ideally suited for the coordination of dorsal and ventral attention networks. However, some authors have ascribed a similar role to the right insular cortex, which they conceive as a ‘switch’ that adapts cortical network activities^33,34^. This proposal is motivated by the involvement of the insula in a ‘saliency network’^34^, which is activated upon the appearance of biologically significant stimuli, in particular when these affect bodily functions^35^. Such partly overlapping conceptions of the role of lPFC and insula are even more complicated by the fact, that the right TPJ has also been conceived as a switch that interrupts processing and terminates the current task set when an unexpected event requires attention^36^. Whether the roles ascribed to these regions are complementary or contradictory is difficult to judge based alone on functional imaging evidence, as cortical networks are highly dynamic and may quickly reconfigure when task demands change^37,38^.

We here studied the processing of task relevance in distinct patient groups with damage to the TPJ or lPFC and insula, and compared them to healthy controls as well as patients with focal subcortical damage. Our assumption was that if a brain region is critical for the computation of task relevance then damage to this region should decrease the liability to orient attention toward behaviorally relevant events. Contrary to this prediction we found that damage to the TPJ, but not the lPFC/insula, pathologically *increases* reflexive attentional capture by task-relevant distracter stimuli.

## Results

**Figure 1:**
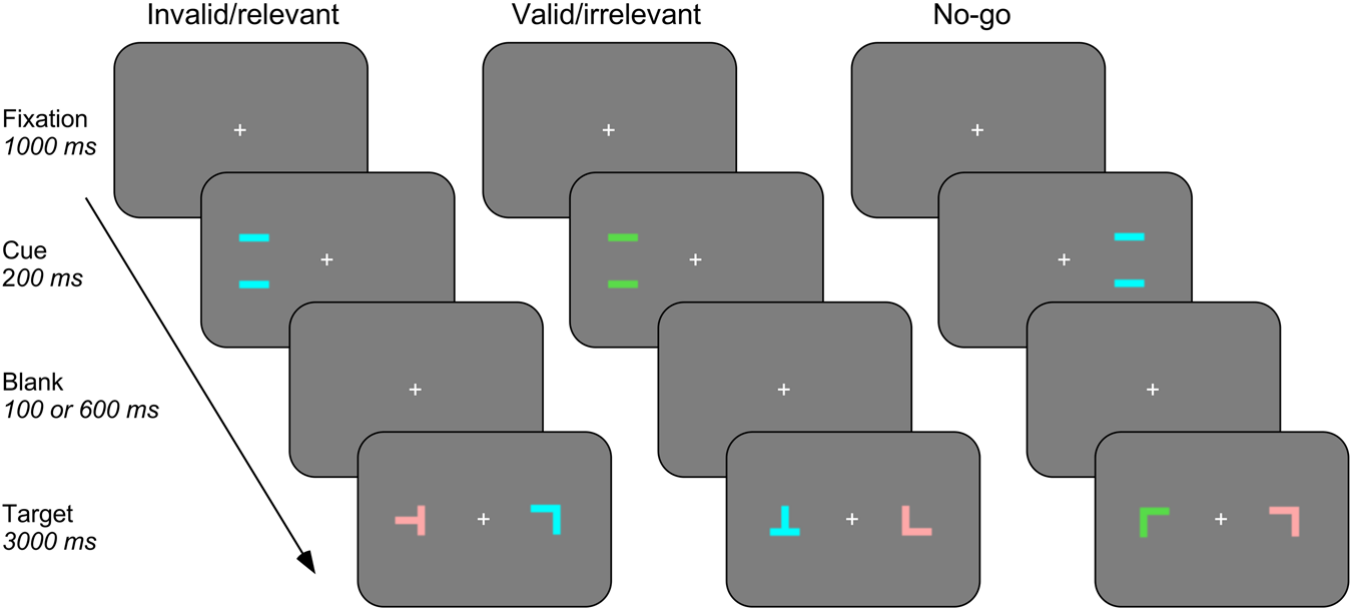
Timeline and examples of different trial types of the spatial cueing task. In all three examples the subject was asked to react to the blue L- or T-shaped target and otherwise to withhold reaction. The examples show different combinations of cue validity, relevance and target position as well as a no-go trial in which participants had to withhold reaction.

**Figure 2:**
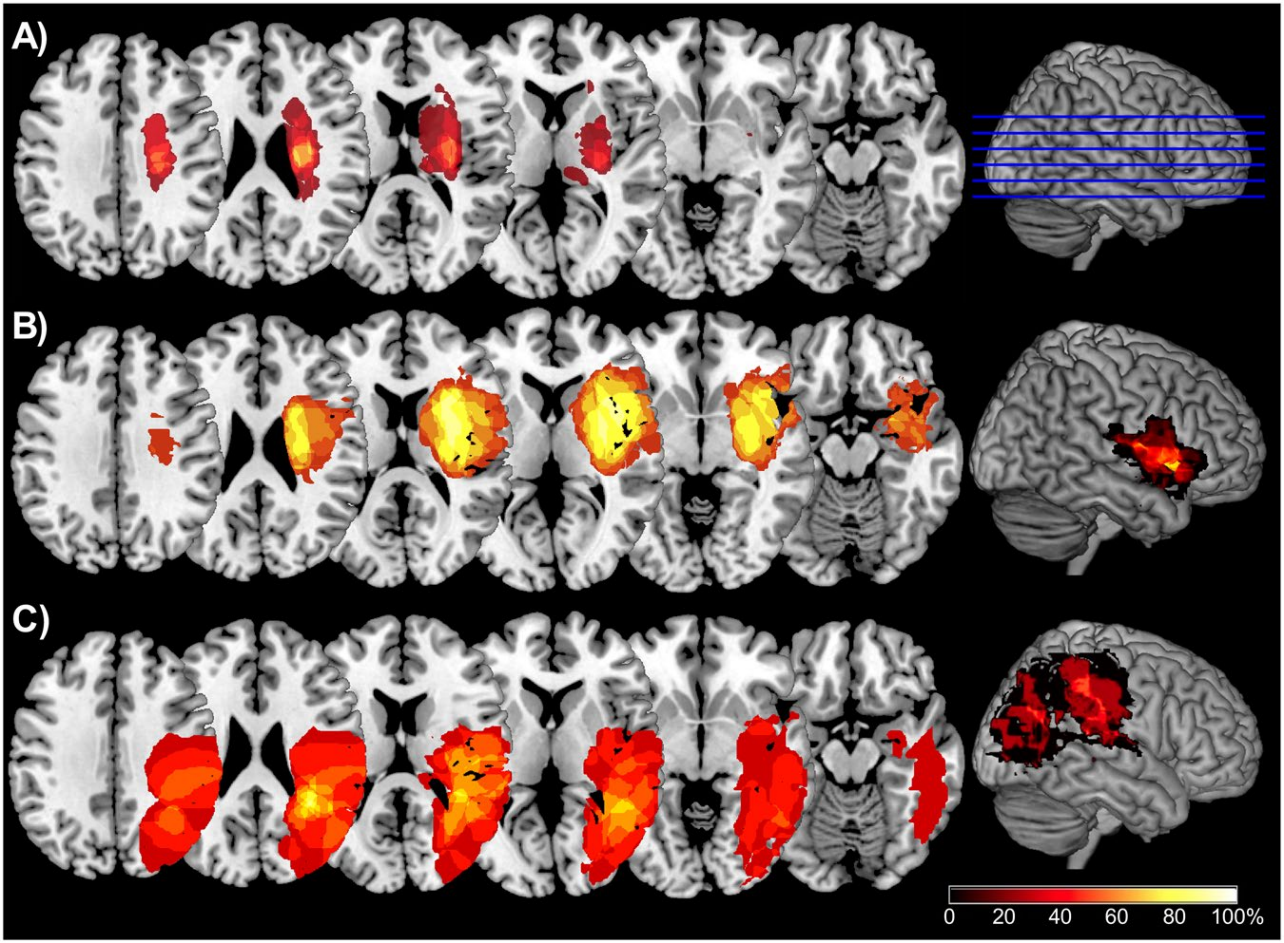
Overlap plots showing axial sections (indicated as blue lines on the 3D-template brain) for (**A**) patients with isolated subcortical damage (SUBCORT group), (**B**) lateral prefrontal and insular cortex damage (lPFC/Ins group) and (**C**) temporoparietal junction damage (TPJ group). Colors represent the percentage of patients sharing damage at a particular location. 3D-plots show the cortical projection of damage extending up to 8 mm subcortically. A region of common overlap of eight TPJ patients is located in the white matter beneath the anterior angular gyrus (MNI-coordinates: 34, −44, 24) and Brodmann area (BA) 39. All 9 patients belonging to the lPFC/Ins group shared a common area of damage in the anterior insular cortex (MNI-coordinates: 40, 6, 6), which overlaps with BA 13. Finally, most patients of the SUBCORT group (7 out of 10) shared damage in periventricular white matter (MNI-coordinates: 23, −13, 23).

We asked patients with acquired damage to the TPJ, the lPFC/Insula, or subcortical white matter (SUBCORT group) and healthy control subjects (CONT group) to react to a colored shape, which was preceded by a brief peripheral cue (Fig. 1). The cue was either in the same hemifield as the target (valid cue) or in the opposite hemifield (invalid cue), and could share the task-relevant color with the target (relevant cue) or had a neutral color (irrelevant cue). The main question was whether the behavioral relevance of distracting cues captures attention in some lesion groups more than in others and whether this effect reflects fast (automatic) or slower (controlled) attentional mechanisms known to manifest themselves differently at short (300 ms) or long (800 ms) stimulus onset asynchronies (SOAs). Figure 2 shows lesion overlap plots of the three patient groups, based on individual MRI scans, normalized and projected on a standard template.

### Omissions and false positive responses

Target misses were very rare in controls (mean omission rate: 0.9%) and rare in patients (4.3%). False positive responses were also rare in all groups (controls: 2.4%; patients: 2.1%). Given that performance was close to ceiling no statistical analysis was performed.

### Reaction time data: between-group comparisons

**Figure 3:**
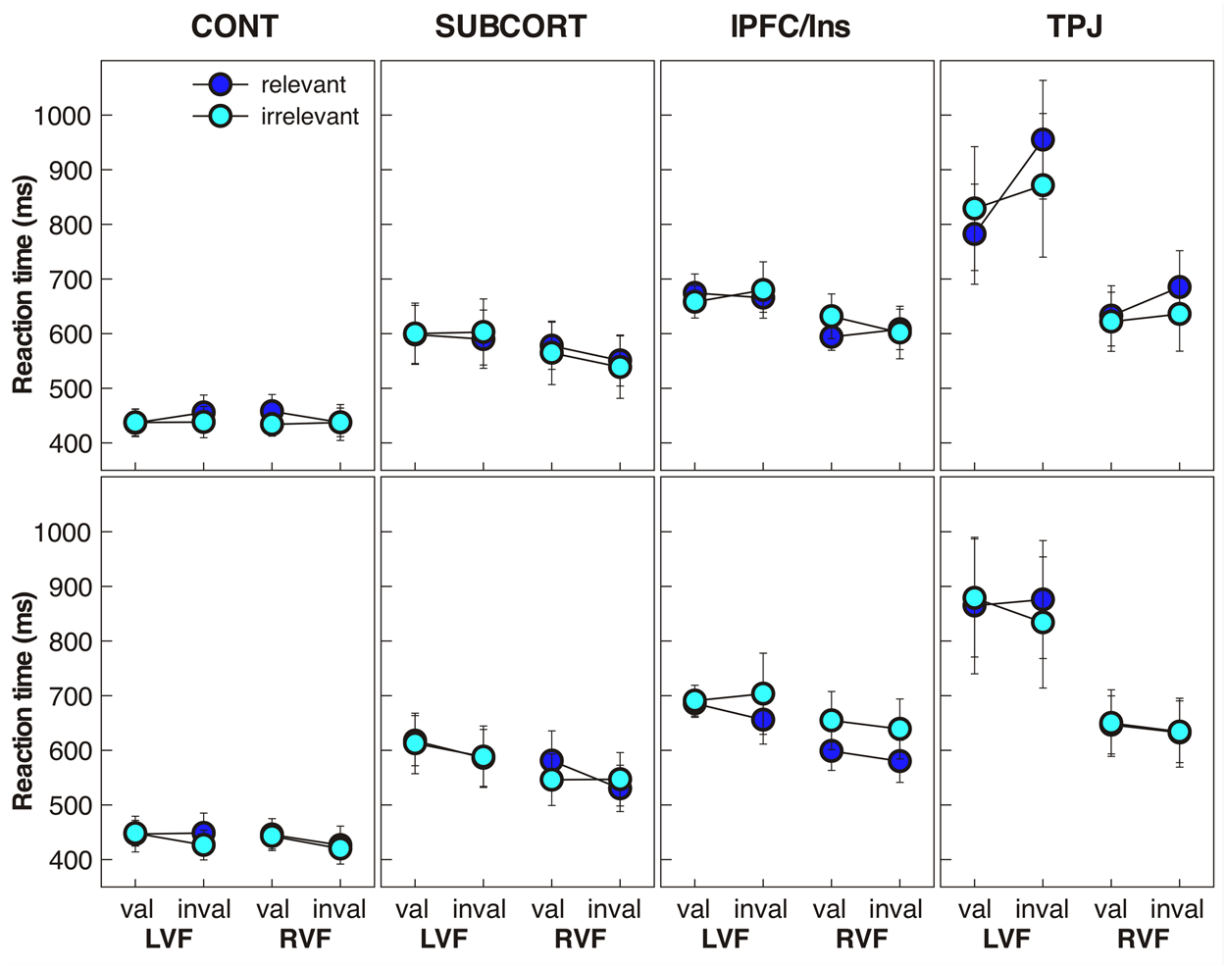
Mean reaction times (±SEM) of the four groups as a function of target position (lvf/rvf: left/right visual field), cue validity and relevance (upper panel: SOA 300; lower panel: SOA 800).

Figure 3 shows the reaction time (RT) data of the four groups. We compared group performance across the repeated factors target position, validity and relevance of the cue in a mixed-model ANOVA (analyses performed with SPSS 25, IBM Inc.).

At short SOAs the analysis revealed significant main effects of group (F_(3,37)_ = 7.19, p = 0.001; ƞ^2^ = 0.368) and target position (F_(1,37)_ = 13.99, p < 0.001; ƞ^2^ = 0.274). Control participants made the fastest reactions (442 ms), followed by SUBCORT (578 ms), lPFC/Ins (639 ms), and TPJ patients (752 ms). The factors group and target position interacted significantly (F_(3,37)_ = 5.14, p = 0.005; ƞ^2^ = 0.294), and both contributed to interaction effects with validity (group: F_(3,37)_ = 5.66, p = 0.003; ƞ^2^ = 0.315; target position: F_(1,37)_ = 5.07, p = 0.030; ƞ^2^ = 0.120). We further found a significant two-way interaction between validity and relevance (F_(1,37)_ = 5.34, p = 0.027; ƞ^2^ = 0.126). Since all of these factors also participated to higher-order effects we did not follow up these interactions. The three-way interaction between group, validity and relevance was significant (F_(3,37)_ = 5.8, p = 0.002; ƞ^2^ = 0.320), but there was yet a higher-order effect as the four-way interaction involving all four factors reached significance (F_(3,37)_ = 5.07, p = 0.005; ƞ^2^ = 0.291). However, to follow up this complex interaction would have required performing a large number of post-hoc tests. Given the presence of important differences in global RT between groups we therefore pursued with a series of simpler repeated-measures ANOVAs that evaluated performance of each group separately (see next section).

A comparable analysis of the RT data at the long SOA yielded a significant effect of group (F_(3,37)_ = 8.03, p < 0.001; ƞ^2^ = 0.394), target position (F_(1,37)_ = 11.16, p = 0.002; ƞ^2^ = 0.232) and validity (F_(1,37)_ = 6.48, p = 0.015; ƞ^2^ = 0.149). Again, controls had the fastest reactions (438 ms), followed by SUBCORT (576 ms), lPFC/Ins (651 ms), and TPJ patients (752 ms). The only other significant effect was a two-way interaction between group and target position (F_(3,37)_ = 3.34, p = 0.030; ƞ^2^ = 0.213), all remaining effects being far from significant (highest F_(3,37)_ = 1.94, p > 0.14).

### Reaction time data: within-group comparisons

The effects of target position, validity and relevance on RTs of each group were analyzed with repeated measures ANOVAs separately for the short and long SOA data. Family-wise error (FWE) for post-hoc tests was controlled through Bonferroni correction. For the CONT group this analysis revealed at the short SOA a significant interaction between target position and cue validity (F_(1,11)_ = 5.66, p = 0.037, ƞ^2^ = 0.340). However, post-hoc tests failed to reach the Bonferroni-corrected significance level. At long SOAs none of the effects and interactions was significant though the effect of target position approached the significance level (F_(1,11)_ = 4.48, p = 0.058, ƞ^2^ = 0.289). Subjects tended to be faster for targets shown in the right hemifield (433 ms) than targets in the left hemifield (443 ms).

At short SOAs the SUBCORT group displayed longer RTs to left (598 ms) than right targets (558 ms; effect of target position: F_(1,9)_ = 15.99, p = 0.003, ƞ^2^ = 0.640), and was marginally faster for targets following invalid (571 ms) than valid cues (585 ms; F_(1,9)_ = 4.74, p = 0.058, ƞ^2^ = 0.345). All other effects and interactions failed to reach significance. At long SOAs the effects were similar, with a significant effect of target position (left: 601 ms; right: 551 ms; F_(1,9)_ = 11.27, p = 0.008, ƞ^2^ = 0.556) and of validity (valid: 589 ms; invalid: 563 ms; F_(1,9)_ = 10.99, p = 0.009, ƞ^2^ = 0.550). While other effects were not significant, we found a significant interaction between validity and relevance (F_(1,9)_ = 9.32, p = 0.014, ƞ^2^ = 0.509). Post-hoc paired t-tests revealed that relevant cues slowed down RTs when they were valid (relevant: 599 ms; irrelevant: 579 ms; t_9_ = 3.43, p = 0.008), but not when they were invalid (relevant: 558 ms; irrelevant: 568 ms; t_9_ = 1.01, p = 0.34). Importantly these effects did not interact with target position and were therefore comparable for the left and right hemifield.

Patients with lPFC/Insula damage displayed significantly longer RTs to left (670 ms) compared to right targets at short SOAs (609 ms; F_(1,8)_ = 28.85, p = 0.001, ƞ^2^ = 0.783). No other effect was significant. At long SOAs the slowing of RTs to left (684 ms) compared to right targets (618 ms) remained significant (F_(1,8)_ = 47.48, p < 0.001, ƞ^2^ = 0.856). Again, no other effect or interaction approached significance. There is no indication in these effects that patients belonging to the lPFC/Ins group attended to relevant cues differently than to irrelevant cues, or that cue relevance interacted with their contralesional slowing of RTs.

Patients with TPJ lesions exhibited a qualitatively distinct pattern of RTs compared to the other patient groups and controls. At short SOAs TPJ patients reacted slower to left (859 ms) than right targets (644 ms; F_(1,9)_ = 6.43, p = 0.032, ƞ^2^ = 0.417). The effect of validity was significant (F_(1,9)_ = 6.05, p = 0.036, ƞ^2^ = 0.402), but there was also a significant interaction between validity and relevance (F_(1,9)_ = 8.69, p = 0.016, ƞ^2^ = 0.491), and more importantly, a significant three-way interaction between target position, validity and relevance (F_(1,9)_ = 5.70, p = 0.041, ƞ^2^ = 0.388). This interaction was due to slower RTs to targets in the left hemifield following an invalid/relevant cue (955 ms) compared to a valid/relevant cue (782 ms; t_9_ = 2.91, p = 0.017) and an invalid/irrelevant cue (872 ms; t_9_ = 2.45, p = 0.037). Thus, behaviorally relevant cues presented in their right hemifield captured attention of TPJ patients and impaired the detection of targets appearing in their left hemifield.

At long SOAs none of these effects were present. We merely observed a marginally significant effect of target position, with slower reactions to left (863 ms) than to right targets (641 ms; F_(1,9)_ = 4.53, p = 0.062, ƞ^2^ = 0.335). All other main effects and interactions were far from approaching the level of significance.

### Validity effects

The analyses of RTs showed that the TPJ group was particularly affected by relevant cues appearing in their right hemifield. However, comparisons of raw RTs may be affected by outliers, in particular exceedingly slow individuals and when small groups are examined. In order to examine whether the relevance effect was disproportionate to the contralesional slowing of TPJ patients we computed a normalized validity effect as follows: VE = (RT_invalid_ − RT_valid_)/(RT_invalid_ + RT_valid_). The resulting index expresses the degree of slowing by invalid cues, relative to global RT, and therefore normalizes differences of basic RT across participants. A positive validity effect reflects slower RTs following invalid cues compared to valid cues (i.e., inhibition), while a negative effect indicates faster RTs (i.e., facilitation).

**Figure 4:**
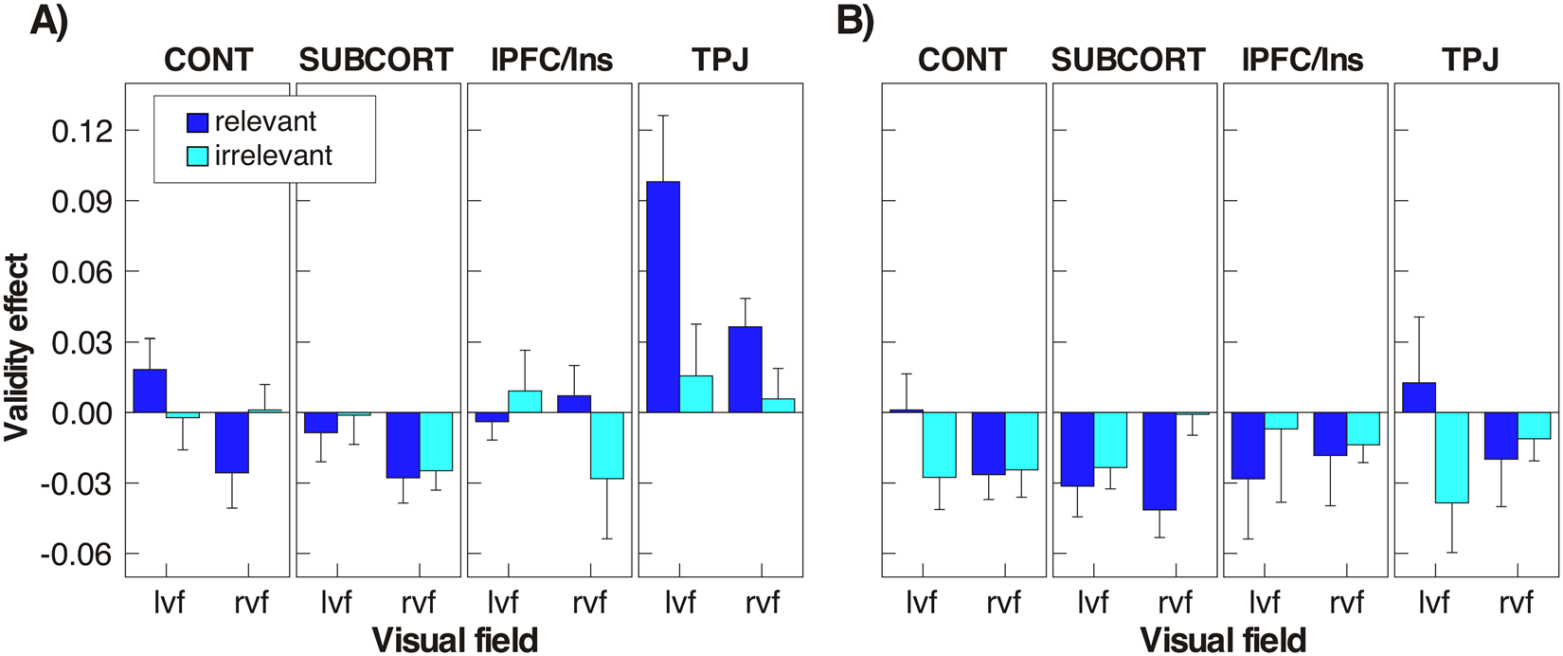
Mean validity effects (±SEM) as a function of target position (lvf/rvf: left/right visual field), cue validity and relevance. A positive validity effect indicates slower RTs following invalid cues compared to valid cues, that is a cost associated with invalid cueing (left panel: SOA 300; right panel: SOA 800).

Figure 4 shows that the validity effects were mostly negative or close to zero, except at short SOAs for the TPJ group. Indeed, at SOA 300 a mixed-measures ANOVA with group as between- and target position and relevance as within-subject factors yielded a significant group effect (F_(3,37)_ = 5.90, p = 0.002, ƞ^2^ = 0.324). Overall, TPJ patients had larger validity effects (0.039) than CONT (−0.002), lPFC/Ins (−0.004) and SUBCORT groups (−0.016). The effect of target position (F_(1,37)_ = 8.08, p = 0.007, ƞ^2^ = 0.179) was also significant, as was the main effect of relevance (F_(1,37)_ = 6.08, p = 0.018, ƞ^2^ = 0.141). We did not follow up these effects as there was a significant interaction between group and relevance (F_(3,37)_ = 5.79, p = 0.002, ƞ^2^ = 0.319) as well as a significant three-way interaction between group, target position and relevance (F_(3,37)_ = 4.33, p = 0.010, ƞ^2^ = 0.260). We performed independent t-tests focusing on comparisons of the TPJ patients with the other three groups. In order to examine the likelihood that non-significant effects are evidence for the null hypothesis of no differences between groups, we also computed the Bayes factor (BF) for t-tests according to previously described methods^39^, implemented in the BayesFactor package for R^40^. A BF ≥ 3 was considered as strong evidence for a true difference between groups, while a BF ≤ 0.3 strongly supports the null hypothesis. As confirmed by inferential and Bayesian statistics relevant cues yielded significantly larger validity effects for left hemifield targets in TPJ patients than CONT (t_20_ = 2.71, p = 0.013, BF = 4.18), SUBCORT (t_18_ = 3.46, p = 0.003, BF = 13.5) and lPFC/Ins groups (t_17_ = 3.23, p = 0.004, BF = 10.17). In contrast, no statistically significant differences were observed for irrelevant cues (all p > 0.49, BF < 0.46). For targets in the right hemifield TPJ patients had larger validity effects to relevant cues than the CONT (t_20_ = 3.13, p = 0.005, BF = 8.26) and the SUBCORT group (t_18_ = 3.94, p = 0.001, BF = 30.93), while the comparison with lPFC/Ins patients was not significant (t_17_ = 1.64, p = 0.12, BF = 0.99). Again, no differences between groups were found for irrelevant cues (all p > 0.13, BF < 0.97), though TPJ patients showed a tendency toward higher indices than SUBCORT patients (t_18_ = 1.97, p = 0.064, BF = 1.45).

At long SOAs the only effect we observed was a significant interaction between group and relevance (F_(3,37)_ = 3.25, p = 0.032, ƞ^2^ = 0.209); however, none of the post-hoc t-tests reached significance (all p > 0.18, BF < 0.80). Together, these findings confirm that even when their slowing of RTs to left hemifield targets was taken into account, TPJ patients exhibited a disproportionate sensitivity to relevant cues.

Finally, given the group differences in age, lesion volume and performance on neglect measures we examined to what extent the latter variables predicted behavior in the experimental task. In a first step we found that lesion volume was a significant predictor of cancellation errors and line bisection bias (Pearson correlation coefficients: r > 0.54, p < 0.003), but not of the validity effect for left or right targets. In a second step we computed correlations between age, neglect measures and validity effects by partialling out the contribution of lesion volume. None of the correlations with age reached significance (r < 0.261), and of the neglect measures only the correlation between line bisection and the validity effect for targets in the left hemifield following relevant cues approached the Bonferroni-corrected significance level (r = 0.43, p = 0.025). Thus, the degree of neglect was at the best a modest predictor of performance in the experimental task.

### Voxel-based lesion-symptom mapping

In order to identify statistically the brain region that is crucial for the observed effects of behavioral relevance we performed voxel-based lesion-symptom (VLSM) mapping^41,42^ using the NiiStat toolbox (https://www.nitrc.org/projects/niistat/). For this analysis voxels damaged in at least 20% of all 29 patients were examined with a general linear model as possible predictors of behavioral performance. The FWE was controlled by adjusting the significance level through permutation testing (4000 permutations).

**Figure 5:**
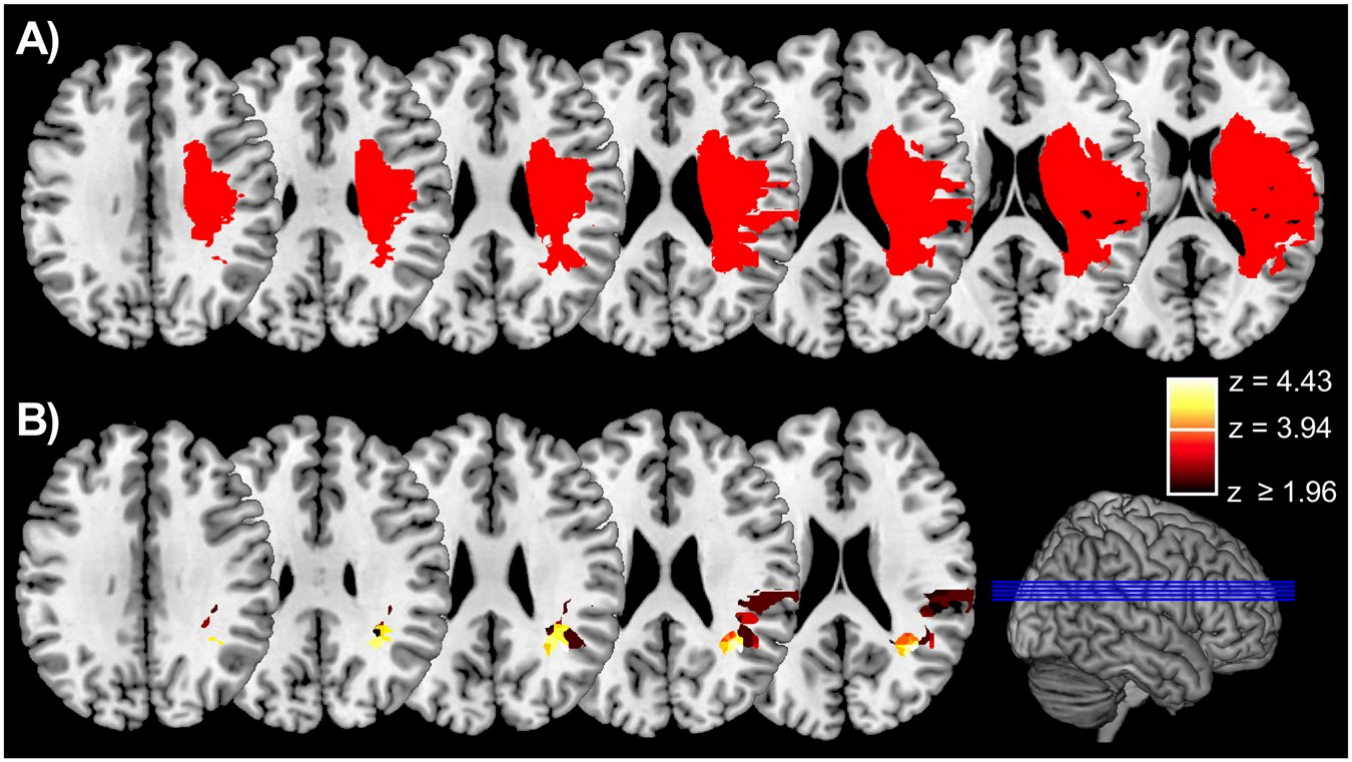
Power and results of the voxel-based lesion-symptom mapping (VLSM) analysis. (**A**) Regions in which statistical power of the VLSM-analyses was at least 80%. (**B**) Axial sections showing voxels predicting the validity index at an uncorrected significance level (p < 0.01) as well as those reaching the FWE-corrected significance threshold (z = 3.94).

We first examined anatomical predictors of the validity effect for irrelevant cues, computed for RT data at the short SOA. Out of 806’580 voxels examined none exceeded the FWE-corrected p-threshold of 0.05. In contrast, the VLSM analysis identified a continuous cluster of 57 voxels lying beneath the anterior angular gyrus as significant predictor of attentional capture by relevant cues (Fig. 5). This region is nearly identical to the maximal overlap of the TPJ group, thus confirming statistically the importance of this area for the exaggerated attention of TPJ patients to behaviorally relevant cues.

**Figure 6:**
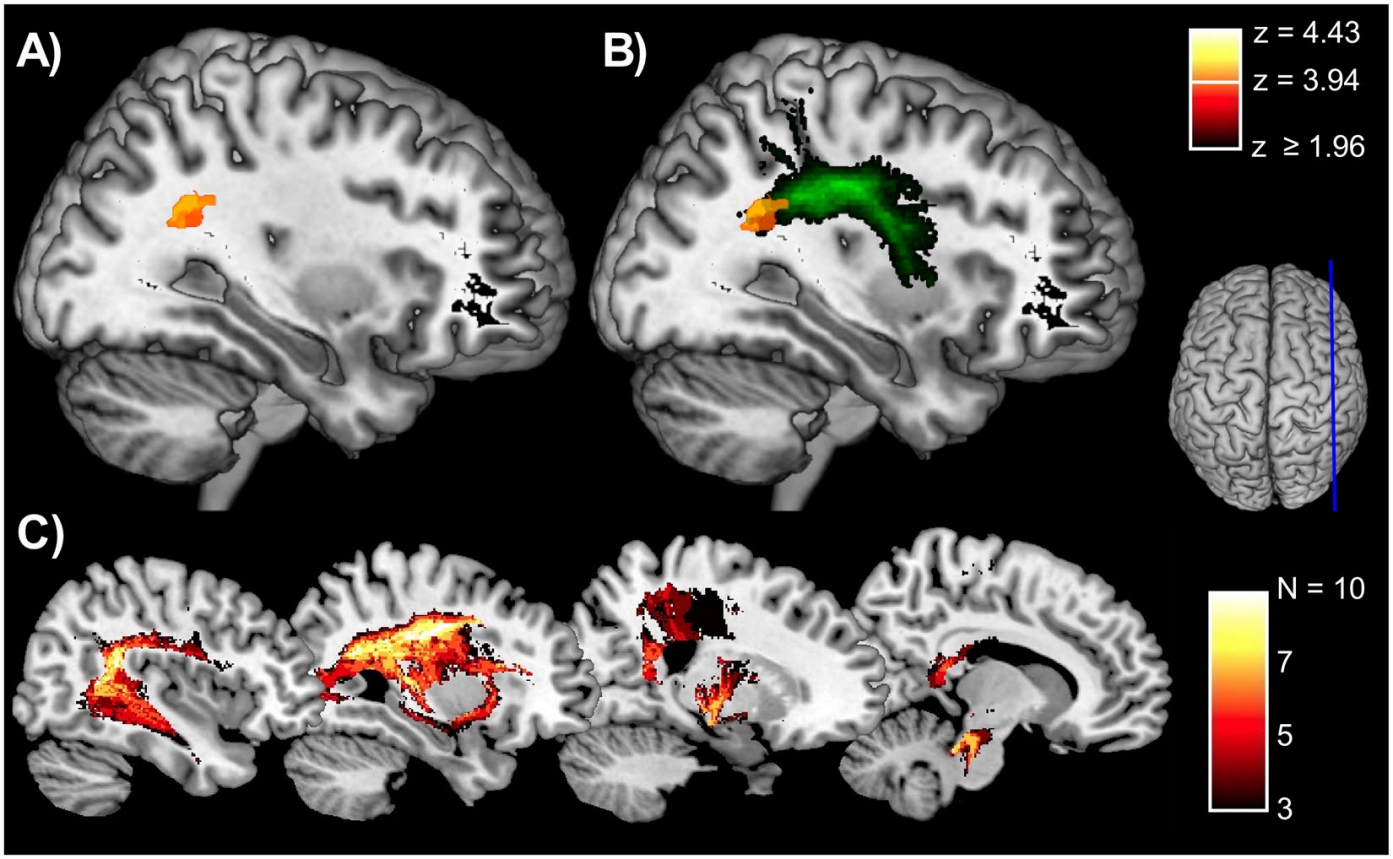
Results of connectivity analysis. (**A**) Sagittal section showing voxels reaching the FWE-corrected significance threshold lying in the white matter beneath the angular gyrus. In (**B**) the same voxels are mapped onto a representation of the superior longitudinal fasciculus (green). (**C**) Probabilistic analysis of disconnection patterns in the TPJ group. The figure colors show the number of patients with disconnection in the visualized areas reaching at least 50% probability.

Given the location of this region in the white matter beneath the angular gyrus, we also examined possible disconnection patterns in the three patient groups. Figure 6 shows that voxels most predictive of the validity effect overlap with the posterior part of the superior longitudinal fasciculus (SLF)^43^. We used the BCBtoolkit (www.toolkit.bcblab.com) to determine how lesions of patients affected fiber tracts extracted from an atlas of healthy subjects^44^ by computing disconnection patterns that would be obtained if the lesion of each patient was taken as seed for probabilistic diffusion tensor tractography^45^. The resulting maps show areas where disconnection for specific fiber tracts passing through the lesion of each patient had a probability of at least 50%. The probability of disconnection was increased in the TPJ group for frontoparietal and frontotemporal fiber tracts, in particular the SLF, the arcuate fasciculus and the fronto-occipital fasciculus (Fig. 6).

## Discussion

The priority that our brain attributes to a particular environmental stimulus depends on multiple factors, such as physical stimulus characteristics, or motivations and expectancies of the observer. Numerous psychophysical studies have shown that perceptual characteristics like stimulus contrast or abrupt appearance capture attention in a very fast and automatic way^24,46–48^. An ongoing controversy is whether these seemingly automatic effects may be overwritten by top-down predispositions^49,50^. Here, we focused on the role of the current task-set and therefore kept constant the bottom-up characteristics, such as luminance, size and contrast of the distracting cues, thus excluding any sensory differences between relevant and irrelevant cues. Nevertheless, the abrupt onset of the cues was a salient event, which should capture attention in a reflexive way. Not surprisingly, we found that acquired lesions of the right subcortical white matter, lPFC/Insula and TPJ diffusely increased RTs to targets in the left hemifield. However, only damage to the TPJ also led to exaggerated capture of attention by behaviorally relevant cues presented in the right hemifield shortly prior to the left hemifield target.

The spatial cueing paradigm employed in this study allows disentangling the potentially interacting effects of target position, validity and cue relevance. The effect of target position was the least informative, as all three patient groups showed significant contralesional slowing of RTs. Slowed reaction to contralesional targets thus appears to be an unspecific consequence of unilateral brain damage and does not depend on the position of the cue or its relevance. In contrast, the validity effect differentiated between groups, as controls and subcortical patients detected targets earlier following invalid than valid cues, lPFC/Insula patients showed no difference, and TPJ patients showed facilitation by valid compared to invalid cues for targets in the left hemifield. Given the absence of positive validity effects in controls one might be tempted to conclude that our task lacks sensitivity to spatial orienting mechanisms. Spatial validity effects strongly depend on reflexive capture of attention, a process that is facilitatory in the first 200–300 ms following cue onset^51^, followed by an inhibitory period that triggers a shift of attention away from the cue (inhibition of return^52^). In previous studies using a similar paradigm^53,54^, we observed in healthy participants faster RTs to valid cues only when the cue was similar to the target, suggesting that positive validity effects reflect contingent attentional capture^55^. Thus, the absence of validity effects in all groups except the TPJ patients at short SOAs might be explained by the length of the SOA (300 ms), which was at the upper limit of the time window of reflexive mechanisms of attention. Previous studies have observed a slowed reorienting response after a right shift of attention in patients with right-hemispheric injury^56,57^. Validity effects have been interpreted as indicators of attentional operations involving disengagement and reorienting^54,58–60^, and deficits of the disengagement from ipsilesional stimuli have been observed in patients with lesions of superior parietal cortex^61,62^, dorsal premotor cortex^60^, or the TPJ^63,64^. Thus, the impact of target validity on performance of TPJ patients is compatible with previous observations. In contrast, though the lPFC and insula have been portrayed as representing an anterior node of the ventral attention network^10,31,32^ damage to these regions did not support any specific role beyond a diffuse contribution to cognitive speed.

The main interest of our study concerns the impact of task-relevant cues on target detection. Neither controls nor patients with subcortical or lPFC/Insula lesions did show any differential reaction to relevant as compared to irrelevant cues. This absence of relevance effects cannot entirely be explained by the choice of specific cue-target intervals since psychophysical studies with healthy subjects have found effects of task-relevance across a broad range of SOAs^55,65^. In order to interpret the attentional processes differentiating TPJ patients from the other groups correctly it is important to comprehend the cognitive demands of the spatial attention task. In our paradigm relevant cues were defined as stimuli possessing a target-defining feature, which could interfere with target detection and whose presence was potentially detrimental for task performance. However, although cues were task-relevant they were not response-relevant, and subjects had to withhold potential answers through adequate control mechanisms. TPJ damage must have impaired the ability to activate such control mechanisms.

Based on these considerations, what part can we conceive for the TPJ in the processing of behaviorally relevant information? The data argue against a role in ascribing behavioral value to specific stimulus features. The main argument against such a role is that TPJ patients must have access to information about task-relevance, otherwise they would not even differentiate between relevant and irrelevant stimuli. If the neural coding of relevance were the domain of the TPJ damage to this region should decrease, rather than overemphasize effects of relevance. Neurophysiological and lesion studies show that the definition of behavioral goals based on specific sensory features relies on the dorsal PFC. Neural responses in frontal areas such as the FEF are sensitive to the similarity between target and distracters^66^, differentiate between target and non-target features earlier than posterior and inferior areas^67^ and directly affect the gain of responses to target features in occipito-temporal cortex^68^. Further, in tasks requiring highly attentive processing of stimulus features the frontal cortex is the first region with access to information distinguishing targets from distracters^69^. These neurophysiological findings are complemented by transcranial magnetic stimulation studies showing that biasing activity of the frontal cortex has distant effects on blood-flow in extrastriate cortex and the TPJ^70^. The analysis of fiber presence in the three lesion groups suggested that patients with TPJ damage have a high probability of disconnection of frontoparietal circuits, in particular the SLF. The SLF is the main fiber tract connecting the inferior and superior parietal lobe with premotor cortex, the lPFC and the insula^71^, and disconnection of this pathway predicts an isolation of the TPJ from modulating prefrontal influence. Though our analysis was not based on direct analysis of disconnection patterns in each individual patient, the findings support the conclusion that signals carrying information about behavioral relevance are not necessarily transmitted directly from the PFC to the TPJ. Rather, we hypothesize that these signals travel along short projections from the posterior parietal cortex^72^, where behavioral goals and sensory features of stimuli are integrated into a priority map of the environment^4,73^. Together, these findings support a top-down organization where target templates for current action goals are defined and maintained in PFC before being relayed to posterior areas such as the intraparietal cortex and TPJ^74,75^. On this view, the TPJ does not directly compute the relevance of a stimulus feature, but modulates its response to stimuli according to-down biasing signals and controls the engagement of attention to potentially distracting, task-related information^18,76^.

In contrast to the TPJ, damage to the lPFC and insular cortex did not affect attentional processing beyond the contralesional slowing of RTs. For both regions this finding is surprising, though not for the same reasons. Based on functional imaging the lPFC has been defined as site of convergence of goal-directed and stimulus-driven signals processed by the dorsal and ventral attention network^32,77^. However, given such a role we would expect damage to this region to affect in some way the balance between processing of relevant and irrelevant cues. There was no evidence for any effect of the cues, and our findings therefore call for further lesion studies evaluating the role of the lPFC in attention paradigms other than spatial cueing tasks.

A different line of reasoning must be put forward for the insula. Together with the anterior cingulate and other limbic and paralimbic cortex the anterior insula is a key part of a ‘saliency network’^34^, whose potential role includes the detection of relevant stimuli across multiple modalities^35^. However, the term saliency is not unequivocal and may refer to perceptual characteristics as well as motivational and affective value of stimuli^78,79^. Lesion studies do not support a direct role of the insula in spatial attention if damage is isolated or when the contributions of TPJ and the parietal cortex are partialled out^80–82^. A distinctive effect of damage to the insular cortex is altered awareness of autonomic responses, visceral experiences and pain, suggesting that the primary role of the insula and other regions of the saliency network has to do with interoceptive signals such as bodily states and hedonic experiences^83,84^. These findings indicate that while the insula is involved in interoceptive attention, orienting of attention toward external stimuli whose relevance is determined by purely cognitive valuation does not depend on this region.

In conclusion, our findings support a decisive role of the TPJ in the orienting of attention toward stimuli whose relevance has been determined by cognitive task sets. We argue that the TPJ receives signals carrying information about current attention settings from regions belonging to the dorsal attention network. The magnified capture of attention observed after TPJ damage suggests that top-down signals arriving at the TPJ are modulated by feedback from the dorsal premotor, prefrontal and possibly the posterior parietal cortex before a shift of attention toward the stimulus with highest current priority is initiated.

## Methods

### Participants

Twenty-nine patients (16 females) with focal right brain lesions following first-ever ischemic stroke were recruited at the Division of neurorehabilitation of the Geneva University Hospitals. Patients were examined at the post-acute stage (≥20 days post-stroke) and were selected based on neuroimaging evidence of focal damage comprising the right TPJ (group TPJ, N = 10) or lPFC/insula (group lPFC/Ins, N = 9). Due to the anatomical arrangement of the vascular tree damage from stroke is rarely confined to cortical regions, and indeed all patients of the TPJ and lPFC/Ins groups had mixed cortico-subcortical lesions. Therefore, in order to control for the contribution of pure subcortical damage to attention capture we additionally recruited a third group of patients with isolated subcortical lesions (group SUBCORT, N = 10). Patients were compared to a group of 12 age-matched healthy controls (group CONT; age, 54.7 ± 3.0 years). The study was approved by the Ethical Commission of the Canton of Geneva, and all participants gave informed consent before being enrolled. The research reported in this study was performed in accordance with relevant guidelines and regulations.

Patients were examined with a battery of standard clinical tests assessing deficits of spatial attention. Tests included the Bells cancellation test^85^, letter cancellation^86^, cancellation of inverted ‘Ts’^87^, line bisection^88^, reading of compound words^89^ and clock drawing^90^. Visual fields were examined in all patients with digital confrontation, and additionally in patients with posterior lesions with dynamic campimetry (detection of running numbers presented in the periphery on a 24” screen while maintaining fixation). As the presence of homonymous impairments of the visual field was an exclusion criterion none of the included patients had a visual field loss.

Table 1 shows demographic and clinical characteristics of all participant groups. Participants’ age differed across groups (*F*_(3,40)_ = 3.29, p = 0.031) because the SUBCORT group was older than the lPFC/Ins group (Bonferroni contrast, p = 0.044; none of the other group comparisons approached significance). Time since stroke was comparable across patient groups (*F*_(2,28)_ = 0.74, p = 0.487), but there was a significant difference regarding lesion volume (*F*_(2,28)_ = 9.17, p = 0.001), which was greater in the TPJ group than the SUBCORT group (p = 0.001), but comparable to the lPFC/Ins group (p = 0.19). ANOVAs comparing patient performance in tests of spatial attention were all significant (lowest *F*_(2,25)_ = 4.13, p < 0.029), except for clock drawing (*F*_(2,22)_ = 0.73, p = 0.493). Patients of the TPJ group had worse performance than the SUBCORT group in all three cancellation tests, on line bisection and in reading (Bonferroni contrasts, all p < 0.032). The TPJ group had also worse performance than the lPFC/Ins group in the T-cancellation test, on line bisection and in reading (p < 0.025). In contrast, none of the comparisons between the lPFC/Ins and SUBCORT group approached significance (all p > 0.5).

**Table 1:** Demographic data (mean ± SEM) and the results of clinical testing of the patient groups (cancellation tests: number omissions on the left side; line bisection: percentage right-sided bias; reading: number of words missed/transformed; clock drawing: number of points given to the drawing. max. 10).

	Age	Time post	Lesion volume (ccm^3^)	Bells cancellation	Letter cancellation	Inverted T cancellation	Line bisection	Reading (of 40)	Clock drawing
subcort1	79.1	41	5.0	1	0	0	0	0	10
subcort2	65.6	88	16.6	0	—	1	7.11	1	10
subcort3	41.1	33	19.7	0	0	1	−0.17	0	9
subcort4	76.1	53	25.5	3	3	3	4.9	20	3
subcort5	70.2	38	1.5	0	0	0	0.23	1	10
subcort6	67.4	48	47.0	6	5	2	1.45	3	10
subcort7	69	34	6.0	0	0	0	0	0	8
subcort8	78.3	42	0.8	2	1	—	—	—	7
subcort9	67.3	39	15.1	11	8	0	0.11	3	3
subcort10	50.1	37	3.0	0	0	0	0.38	2	—
*SUBCORT*	*66.4* ± *3.8*	*45.3* ± *5.1*	*14.0* ± *4.5*	*2.3* ± *1.2*	*0.8* ± *0.4*	*1.9* ± *1.0*	*1.6* ± *0.9*	*3.3* ± *2.1*	*7.8* ± *1.0*
lpfc/ins1	81.3	35	24.5	1	0	0	−0.28	1	10
lpfc/ins2	33.6	29	132.5	2	2	0	0	0	9
lpfc/ins3	49.3	20	34.6	0	2	2	0	0	10
lpfc/ins4	41.1	40	106.2	7	4	3	2.45	4	—
lpfc/ins5	28.5	82	51.9	2	0	0	2.73	5	8
lpfc/ins6	48.7	90	106.8	5	6	3	−1.37	4	—
lpfc/ins7	62.3	49	62.9	7	6	4	3.96	6	—
lpfc/ins8	55.7	29	54.7	2	3	2	0.78	2	9
lpfc/ins9	48.6	32	69.4	4	5	4	−0.11	—	—
*lPFC/Ins*	*49.9* ± *5.2*	*45.1* ± *8.2*	*71.5* ± *12.1*	*3.3* ± *0.8*	*2.0* ± *0.6*	*3.1* ± *0.8*	*0.9* ± *0.6*	*2.8* ± *0.8*	*9.2* ± *0.4*
tpj1	36	32	184.7	15	10	—	8.78	20	10
tpj2	70	98	38.2	5	6	3	3.45	1	10
tpj3	64.7	41	158.0	15	20	17	12.76	24	2
tpj4	61	27	95.2	1	2	0	10.8	7	10
tpj5	69.5	45	84.9	10	21	11	5.79	19	7
tpj6	43.8	130	142.6	14	26	—	17.03	29	—
tpj7	69.3	89	316.8	11	18	9	9.82	2	8
tpj8	63.4	41	64.3	3	5	4	2.44	4	2
tpj9	77	43	4.84	3	2	1	9.52	8	10
tpj10	65.1	32	126.4	2	8	0	6.7	11	7
*TPJ*	*61.6* ± *4.4*	*60.7* ± *12.0*	*121.6* ± *27.8*	*7.9* ± *1.8*	*5.6* ± *2.2*	*11.8* ± *2.8*	*8.7* ± *1.4*	*12.4* ± *3.1*	*7.3* ± *1.1*

### Experimental Design

Participants completed a spatial cueing task that required them to detect and react to a target stimulus following a peripheral cue. The apparatus and stimuli were identical to a previous study examining electrophysiological correlates of attentional capture by task-relevant stimuli^24^, though the task was different. The stimuli were presented on a 21-inch CRT screen (refreshed at 85 Hz) at 60 cm distance from de patient, and reaction times (RTs) were measured directly on the keyboard. Target and distracter stimuli were composed of two colored elements arranged in a 3° × 3° square so that they either formed an L- or a T-shape, and cues were formed by two horizontal bars 4.5 degrees apart (Fig. 1). Targets, distracters and cue were red (RGB-values: 222, 80, 80), green (0, 180, 0) or blue (10, 150, 250), their luminance being comparable (25 cd/m^2^), but slightly higher than the gray background (15 cd/m^2^). In order to avoid that participants treated targets and distracters as letters (which may have activated left-hemisphere mechanisms), we presented them randomly upright or rotated by 90, 180, or 270 degrees. For each participant one color was randomly selected as the target color at the beginning of the session.

A trial began with the onset of a central fixation cross for 1000 ms, followed by a single colored cue at a peripheral position (5° left or right of fixation) that remained on the screen for 200 ms (Fig. 1). After a blank interval of 100 or 600 ms, the target display (consisting of two colored shapes (either a target and a distracter or two distracters) appeared. One of the two shapes appeared at the previous position of the cue and the other at a corresponding position in the opposite visual field. We preferred to present the target simultaneously with a contralateral distracter (rather than alone as in several previous spatial cueing studies) in order to increase attentional competition in the task. The stimulus-onset asynchrony (SOA) between cue and target was either 300 ms or 800 ms, corresponding to distinct periods of automatic and voluntary orienting of attention^52^. Participants were instructed not to react during the presentation of the cue, but to look at the screen attentively and to prepare for the appearance of the target display. They were asked to press the space bar with their right index finger upon the appearance of the target display if one of the two shapes was painted in the target color (go trial) or to withhold reaction if the target color was not present (no-go trial). In valid trials, the target was shown at the same position as the cue while on invalid trials cue and target were presented on opposite sides. In relevant trials cue and target shared the same color while in irrelevant trials, their colors were different. The target display remained on the screen until a button press took place or for 3000 ms. During a practice block participants were given feedback about any commission error made and if necessary reminded to retain reactions until the apparition of the target display.

The main factor of interest was the speed of target detection following presentation of valid or invalid cues, as well as the interaction of this effect with relevance. The four experimental factors target position (left, right), cue validity (valid, invalid), cue relevance (relevant or irrelevant) and SOA (300 ms, 800 ms) were orthogonally varied in blocks of 96 trials containing 64 go and 32 no-go trials. Every participant performed 8 blocks for a total of 768 trials, resulting in 32 go trials and 16 no-go trials for each of the 16 conditions.

### MRI acquisition and analysis

Patients underwent structural magnetic resonance imaging (MRI), including T1-, T2- and diffusion-weighted acquisitions with a between-slice resolution of 4 mm, on a 3 T Trio scanner (Siemens Medical Solutions, Erlangen, Germany). For most patients a standard clinical scanning protocol was applied, but for some patients high-resolution scans were accessible (1.1 mm between-slice resolution). One patient had a metallic clip and neuroimaging was therefore based on a CT scan. Brain lesions were manually outlined on a T2-MRI scan or on the CT scan using MRIcron software (https://www.nitrc.org/projects/mricron) and a graphic tablet. The T2-scans and the lesion volume of interest (VOI) were then coregistered with the T1-weighted image, and each MRI and CT scan including the volume of interest (VOI) matching to the lesion was normalized to standard space using SPM12 (http://www.fil.ion.ucl.ac.uk/spm). Brain normalization was conducted using the Clinical Toolbox^91^, which offers age-specific CT and MRI templates and automatically applies enantiomorphic lesion masking to minimize normalization artifacts induced by abnormal tissue^92^.

## Data Availability

All data generated during this study are available from the corresponding author on request.
